# Supplementary material for: The Evolution and Characterization of the RNA Interference Pathways in Lophotrochozoa
Source: Genome Biol Evol. 2024 May 7;16(5):evae098. doi: 10.1093/gbe/evae098 (PMC11114477; doi:10.1093/gbe/evae098)
Supplement: evae098_Supplementary_Data [file evae098_supplementary_data.zip › Supplementary_materials.zip!/selected_miRNAs/NoGe_selected_miRNAs/mirdeep_pdf_files/NMRB01003740.1_Notospermus_geniculatus_strain_Ushimado_scaffold3740,_whole_genome_shotgun_sequence_44401.pdf]

Provisional ID	:	NMRB01003740.1_Notospermus_geniculatus_strain_Ushimado_scaffold3740,_whole_genome_shotgun_sequence_44401
Score total	:	156820
Score for star read(s)	:	3.9
Score for read counts	:	156811.6
Score for mfe	:	1.4
Score for randfold	:	0
Score for cons. seed	:	3
Total read count	:	307591
Mature read count	:	306064
Loop read count	:	0
Star read count	:	1527

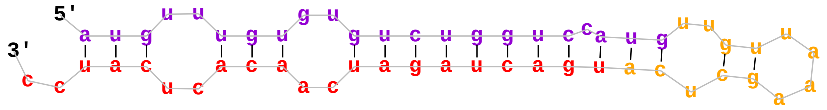

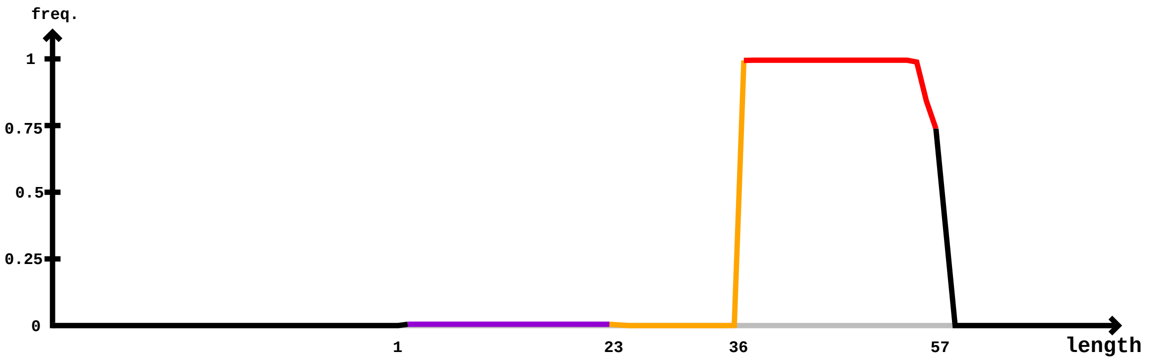


```
                                      Star                                 Mature
5'- cauucaacaugggcagugcagugccagucuuuaggauguuugugugucugguccauguuguuaaagcucaugacuagaucaacacucauccugacggcuuggaaucauc   -3'   obs
    cauucaacaugggcagugcagugccagucuuuaggauguuugugugucugguccauguuguuaaagcucaugacuagaucaacacucauccugacggcuuggaaucauc         exp
    ..........((((......))))((((.((((((((..(((..((((((((.(((..((....)).)))))))))))..)))..)))))))).))))...........   reads   mm   sample
    ...................................auguuugugugucugguGc.......................................................   1       1    Mm3
    ...................................auguuugugugucugguAcaug....................................................   1       1    Mm3
    ...................................auguuugugugucugguGcaug....................................................   1       1    Mm3
    ...................................auguuugugugucugguccaug....................................................   61      0    Mm3
    ...................................auguuugugugucugguccaugu...................................................   2       0    Mm3
    ...................................auguuugugugucugguccauguugu................................................   1       0    Mm3
    .....................................................................augacuagaucCacacucauc...................   2       1    Mm3
    .....................................................................augacuagaucCacacucaucc..................   8       1    Mm3
    ......................................................................ugacuagaucCacacuca.....................   30      1    Mm3
    ......................................................................ugacuagaucCacacucau....................   235     1    Mm3
    ......................................................................ugacuagaucaacacucau....................   1       0    Mm3
    ......................................................................ugacuagaucCacacucauc...................   3336    1    Mm3
    ......................................................................ugacuagaucGacacucauc...................   8       1    Mm3
    ......................................................................ugacuagaucaacacucauc...................   4       0    Mm3
    ......................................................................ugacuagaucCacacucaucc..................   31930   1    Mm3
    ......................................................................ugacuagaucGacacucaucc..................   51      1    Mm3
    ......................................................................ugacuagaucaacacucaucc..................   52      0    Mm3
    ......................................................................ugacuagaucUacacucauccu.................   184     1    Mm3
    ......................................................................ugacuagaucaacacucauccu.................   3       0    Mm3
    ......................................................................ugacuagaucCacacucauccu.................   2807    1    Mm3
    ......................................................................ugacuagaucGacacucauccu.................   4       1    Mm3
    .......................................................................gacuagaucCacacucau....................   1       1    Mm3
    .......................................................................gacuagaucCacacucauc...................   6       1    Mm3
    .......................................................................gacuagaucCacacucaucc..................   35      1    Mm3
    .......................................................................gacuagaucCacacucauccu.................   5       1    Mm3
    .........................................................................cuagaucCacacucaucc..................   1       1    Mm3

    ...................................auguuugugugucuggucca......................................................   9       0    Fm3
    ...................................auguuugugugucugguGca......................................................   1       1    Fm3
    ...................................auguuugugugucugguccau.....................................................   6       0    Fm3
    ...................................auguuugugugucugguccaug....................................................   555     0    Fm3
    ...................................auguuugugugucuggCccaug....................................................   3       1    Fm3
    ...................................auguuugugugucugguccauA....................................................   1       1    Fm3
```

Star **Mature**

cauucaacaugggcagugcagugccagucuuuaggauguuugugugucugguccauguuguuaaagcucaugacuagaucaacacucauccugacggcuuggaaucauc			
...................................auguuugugugucuggGccaug..	2	1	Fm3
...................................auguuuguguAucugguccaug..	4	1	Fm3
...................................auguuugugugucugguGcaug..	1	1	Fm3
...................................auguuuguguUucugguccaug..	1	1	Fm3
...................................auguuuguUugucugguccaug..	1	1	Fm3
...................................auguuugugugucugguUcaug..	4	1	Fm3
...................................auguuugugugucugAuccaug..	1	1	Fm3
...................................auguuugugugucugguAcaug..	7	1	Fm3
...................................auguuugugugucuggAccaug..	1	1	Fm3
...................................auguuugugugucugguccaugA...	2	1	Fm3
...................................auguuugugugucugguccaugu...	1	0	Fm3
...................................auguuugugugucugguccauguu..	2	0	Fm3
...................................auguuugugugucugguccauguug...	2	0	Fm3
...................................auguuugugugucugguccauguugu..	1	0	Fm3
....................................uguuugugugucuggucUaug..	1	1	Fm3
....................................uguuugugugucuCguccaug..	6	1	Fm3
....................................uguuugugugCcugguccaug..	1	1	Fm3
....................................uguuugugugucugguccaug..	10	0	Fm3
....................................uguuugugugucugguccaugu...	2	0	Fm3
....augacuagaucCacacucau.....................	26	1	Fm3
....augacuagaucCacacucauc....................	4	1	Fm3
....augacuagaucCacacucaucc...................	40	1	Fm3
....augacuagaucUacacucauccu.................	1	1	Fm3
....ugacuagaucaacacuca.....................	2	0	Fm3
....ugacuagaucCacacuca.....................	583	1	Fm3
....ugacuagaucGacacucau....................	60	1	Fm3
....ugacuagaucaacacucau....................	64	0	Fm3
....ugacuagaucCacacucau....................	38247	1	Fm3
....ugacuagaucaacacucauc...................	18	0	Fm3
....ugacuagaucCacacucauc...................	7777	1	Fm3
....ugacuagaucaacacucauU...................	1	1	Fm3
....ugacuagaucGacacucauc...................	11	1	Fm3
....ugacuagaucGacacucaucc..................	187	1	Fm3
....uAacuagaucaacacucaucc..................	1	1	Fm3
....ugacuagaucCacacucaucc..................	109454	1	Fm3
....ugacuagaucaacacucaucc..................	200	0	Fm3
....ugacuagaucUacacucauccu.................	884	1	Fm3
....ugacuagaucaacacucauccu.................	12	0	Fm3
....ugacuagaucCacacucauccu.................	6735	1	Fm3
....ugacuagaucGacacucauccu.................	15	1	Fm3
....ugacuagaucCacacucauccug................	5	1	Fm3
....gacuagaucCacacucau.....................	38	1	Fm3
....gacuagaucCacacucauc....................	6	1	Fm3
....gacuagaucCacacucaucc...................	118	1	Fm3
....gacuagaucUacacucauccu..................	2	1	Fm3
....gacuagaucCacacucauccu..................	6	1	Fm3
....acuagaucCacacucaucc...................	5	1	Fm3
...................................auguuugugugucugguc..	1	0	Mm2
...................................Uuguuugugugucugguccaug..	1	1	Mm2
...................................auguuugugugucugguccaug..	40	0	Mm2
...................................auguuugugugucugguccaugu...	1	0	Mm2
....augacuagaucCacacucau.....................	1	1	Mm2
....augacuagaucCacacucaucc...................	6	1	Mm2
....ugacuagaucCacacuca.....................	48	1	Mm2
....ugacuagaucaacacucau....................	6	0	Mm2
....ugacuagaucGacacucau....................	4	1	Mm2
....ugacuagaucCacacucau....................	2319	1	Mm2
....ugacuagaucCacacucauc...................	765	1	Mm2
....ugacuagaucGacacucauc...................	3	1	Mm2
....ugacuagaucaacacucauc...................	1	0	Mm2
....ugacuagaucCacacucaucc..................	12824	1	Mm2
....ugacuagaucaacacucaucc..................	22	0	Mm2
....ugacuagaucGacacucaucc..................	28	1	Mm2
....uAacuagaucaacacucaucc..................	1	1	Mm2
....ugacuagaucUacacucauccu.................	66	1	Mm2
....ugacuagaucCacacucauccu.................	591	1	Mm2
....ugacuagaucaacacucauccu.................	2	0	Mm2
....ugacuagaucGacacucauccu.................	1	1	Mm2
....ugacuagaucCacacucauccug................	1	1	Mm2

```
                                   Star                               Mature
cauucaacaugggcagugcagugccagucuuuaggauguuugugugucugguccauguuguuaaaagcucaugacuagaucaacacucauccugacggcuuggaaucauc
........................................................................gacuagaucCacacucaucc..................  9      1   Mm2

...................................auguuugugugucugguAcaug.....................................................  1      1   Mm1
...................................auguuuguguCucugguccaug.....................................................  1      1   Mm1
...................................auguuugugugucuggAccaug.....................................................  1      1   Mm1
...................................Guguuugugugucugguccaug.....................................................  1      1   Mm1
...................................auguuugugugucugguccaug.....................................................  134    0   Mm1
...................................auguuugugugucugguUcaug.....................................................  1      1   Mm1
......................................................................augacuagaucCacacucauc...................  20     1   Mm1
......................................................................augacuagaucCacacucaucc..................  10     1   Mm1
.......................................................................ugacuagaucGacacuca.....................  1      1   Mm1
.......................................................................ugacuagaucaacacuca.....................  1      0   Mm1
.......................................................................ugacuagaucCacacuca.....................  1309   1   Mm1
.......................................................................ugacuagaucCacacucau....................  1692   1   Mm1
.......................................................................ugacuagaucaacacucau....................  2      0   Mm1
.......................................................................ugacuagaucGacacucau....................  4      1   Mm1
.......................................................................ugacuagaucaacacucauc...................  12     0   Mm1
.......................................................................ugacuagaucGacacucauc...................  22     1   Mm1
.......................................................................ugacuagaucCacacucauc...................  13643  1   Mm1
.......................................................................ugCcuagaucaacacucauc...................  1      1   Mm1
.......................................................................ugacuagaucGacacucaucc..................  32     1   Mm1
.......................................................................ugacuagaucCacacucaucc..................  26840  1   Mm1
.......................................................................ugacuagaucaacacucaucc..................  47     0   Mm1
.......................................................................ugacuagaucCacacucauccu.................  1161   1   Mm1
.......................................................................ugacuagaucUacacucauccu.................  158    1   Mm1
.......................................................................ugacuagaucaacacucauccu.................  3      0   Mm1
.......................................................................ugacuagaucGacacucauccu.................  2      1   Mm1
........................................................................gacuagaucCacacucauc...................  14     1   Mm1
........................................................................gacuagaucCacacucaucc..................  14     1   Mm1

...................................auguuugugugucugguc.........................................................  1      0   Fm1
...................................auguuugugugucugguAcaug.....................................................  2      1   Fm1
...................................auguuugugugucuggGccaug.....................................................  2      1   Fm1
...................................auguuugugugucugguccaGg.....................................................  1      1   Fm1
...................................auguuugugugucugguccaug.....................................................  219    0   Fm1
...................................auguuugugugucuggucUaug.....................................................  1      1   Fm1
...................................auguuugugugucuggCccaug.....................................................  1      1   Fm1
...................................auAuuugugugucugguccaug.....................................................  1      1   Fm1
...................................auguuugugugucugguUcaug.....................................................  1      1   Fm1
...................................auguuugugugucugguGcaug.....................................................  1      1   Fm1
...................................auguuugugugucugguccaugA....................................................  1      1   Fm1
......................................................................augacuagaucCacacucauc...................  10     1   Fm1
......................................................................augacuagaucCacacucaucc..................  3      1   Fm1
.......................................................................ugacuagaucGacacuca.....................  1      1   Fm1
.......................................................................ugacuagaucCacacuca.....................  104    1   Fm1
.......................................................................ugacuagaucaacacucau....................  2      0   Fm1
.......................................................................ugacuagaucCacacucau....................  1663   1   Fm1
.......................................................................ugacuagaucGacacucau....................  3      1   Fm1
.......................................................................ugacuagaucCacacucauc...................  2197   1   Fm1
.......................................................................ugacuagaucGacacucauc...................  2      1   Fm1
.......................................................................ugacuagaucaacacucauc...................  7      0   Fm1
.......................................................................ugacuagaucGacacucaucc..................  21     1   Fm1
.......................................................................ugacuagaucaacacucaucc..................  23     0   Fm1
.......................................................................ugacuagaucaUcacucaucc..................  1      1   Fm1
.......................................................................ugacuagaucCacacucaucc..................  13070  1   Fm1
.......................................................................ugacuagaucUacacucauccu.................  174    1   Fm1
.......................................................................ugacuagaucGacacucauccu.................  1      1   Fm1
.......................................................................ugacuagaucCacacucauccu.................  613    1   Fm1
.......................................................................ugacuagaucaacacucauccG.................  1      1   Fm1
.......................................................................ugacuagaucaacacucauccu.................  4      0   Fm1
.......................................................................ugacuagaucCacacucauccug................  1      1   Fm1
........................................................................gacuagaucCacacucauc...................  1      1   Fm1
........................................................................gacuagaucCacacucaucc..................  5      1   Fm1
........................................................................gacuagaucCacacucauccu.................  1      1   Fm1

...................................auguuugugugucuggCccaug.....................................................  1      1   Fm2
...................................Uuguuugugugucugguccaug.....................................................  2      1   Fm2
...................................auguuugugugucugguGcaug.....................................................  2      1   Fm2
...................................aCguuugugugucugguccaug.....................................................  1      1   Fm2
...................................auguuugugugucugguUcaug.....................................................  2      1   Fm2
```

```
                                   Star                              Mature
cauucaacaugggcagugcagugccagucuuuaggauguuugugugucugguccauguuguuaaagcucaugacuagaucaacacucauccugacggcuuggaaucauc
...................................auguuugugugucugguccaGg....................................................  4      1  Fm2
...................................auguuuguguAucugguccaug....................................................  1      1  Fm2
...................................auguuugugugucugguccaug....................................................  403    0  Fm2
...................................auguuuguguCucugguccaug....................................................  1      1  Fm2
...................................auguuugugugucuggGccaug....................................................  1      1  Fm2
...................................auguuugugugucugguAcaug....................................................  1      1  Fm2
...................................auguuugCgugucugguccaug....................................................  1      1  Fm2
...................................auguuugugugucuggucUaug....................................................  1      1  Fm2
...................................auguuugugugucugguccaugA...................................................  1      1  Fm2
.....................................................................ugacuagaucCacacuca.....................  30     1  Fm2
.....................................................................ugacuagaucCacacucau....................  312    1  Fm2
.....................................................................ugacuagaucaacacucau....................  1      0  Fm2
.....................................................................ugacuagaucCacacucauc...................  4299   1  Fm2
.....................................................................ugacuagaucGacacucauc...................  12     1  Fm2
.....................................................................ugacuUgaucaacacucauc...................  1      1  Fm2
.....................................................................ugacuagaucaacacucauc...................  10     0  Fm2
.....................................................................ugacuagaucCacacucaucc..................  17744  1  Fm2
.....................................................................ugacCagaucaacacucaucc..................  1      1  Fm2
.....................................................................ugacuagaucaacacucaucc..................  33     0  Fm2
.....................................................................ugacuagaucGacacucaucc..................  21     1  Fm2
.....................................................................ugacuagaucCacacucauccu.................  681    1  Fm2
.....................................................................ugacuagaucUacacucauccu.................  109    1  Fm2
.....................................................................ugacuagaucCacacucauccug................  5      1  Fm2
.....................................................................ugacuagaucaacacucauccug................  1      0  Fm2
......................................................................gacuagaucCacacucaucc..................  4      1  Fm2
.......................................................................acuagaucCacacucauc...................  1      1  Fm2
```
